# Neurodevelopmental Abnormalities in Down Syndrome: Assessing Structural and Functional Deficits

**DOI:** 10.7759/cureus.76156

**Published:** 2024-12-21

**Authors:** Joelle Robinson, Nidhi Chawla, Shreya Patel, Eliana Spey, Olivia McNulty, Gurjinder Kaur

**Affiliations:** 1 Department of Physiology, Touro College of Osteopathic Medicine, Middletown, USA

**Keywords:** down syndrome, intellectual disability, motor deficits, neurodevelopmental abnormalities, neurotransmitters, nmr spectroscopy, trisomy 21

## Abstract

Down syndrome (DS) is a genetic intellectual disorder caused by trisomy of chromosome 21 (Hsa21) and presents with a variety of phenotypes. The correlation between the chromosomal abnormality and the resulting symptoms is unclear, partly due to the spectrum of impairments observed. However, it has been determined that trisomy 21 contributes to neurodegeneration and impaired neurodevelopment resulting from decreased neurotransmission, neurogenesis, and synaptic plasticity. DS is linked to synaptic abnormalities and hindered hippocampal neuron development as well. Altered synaptic plasticity in the hippocampus decreases long-term potentiation, leading to short- and long-term learning and memory deficits. Individuals with DS show reduced gray matter, which affects cerebral cortex structure and impairs coordination and thought. Neurotransmitter excess, such as increased gamma-aminobutyric acid (GABA) release, causes over-inhibition and contributes to cognitive deficits. This inhibition also affects hippocampal synaptic plasticity. Additionally, DS often involves neurodegeneration of cholinergic neurons in the basal forebrain, further impairing learning and memory. Reduced glutamate transmission and decreased amyloid precursor protein metabolism contribute to synaptic plasticity deficits and behavioral changes in DS. Decreased neurotransmission, diminished motor neurons, and impaired cerebellar and cerebral development are the main causes of motor deficits in DS. This review discusses the stark structural changes in DS and their functional consequences.

## Introduction and background

**Figure 1:**
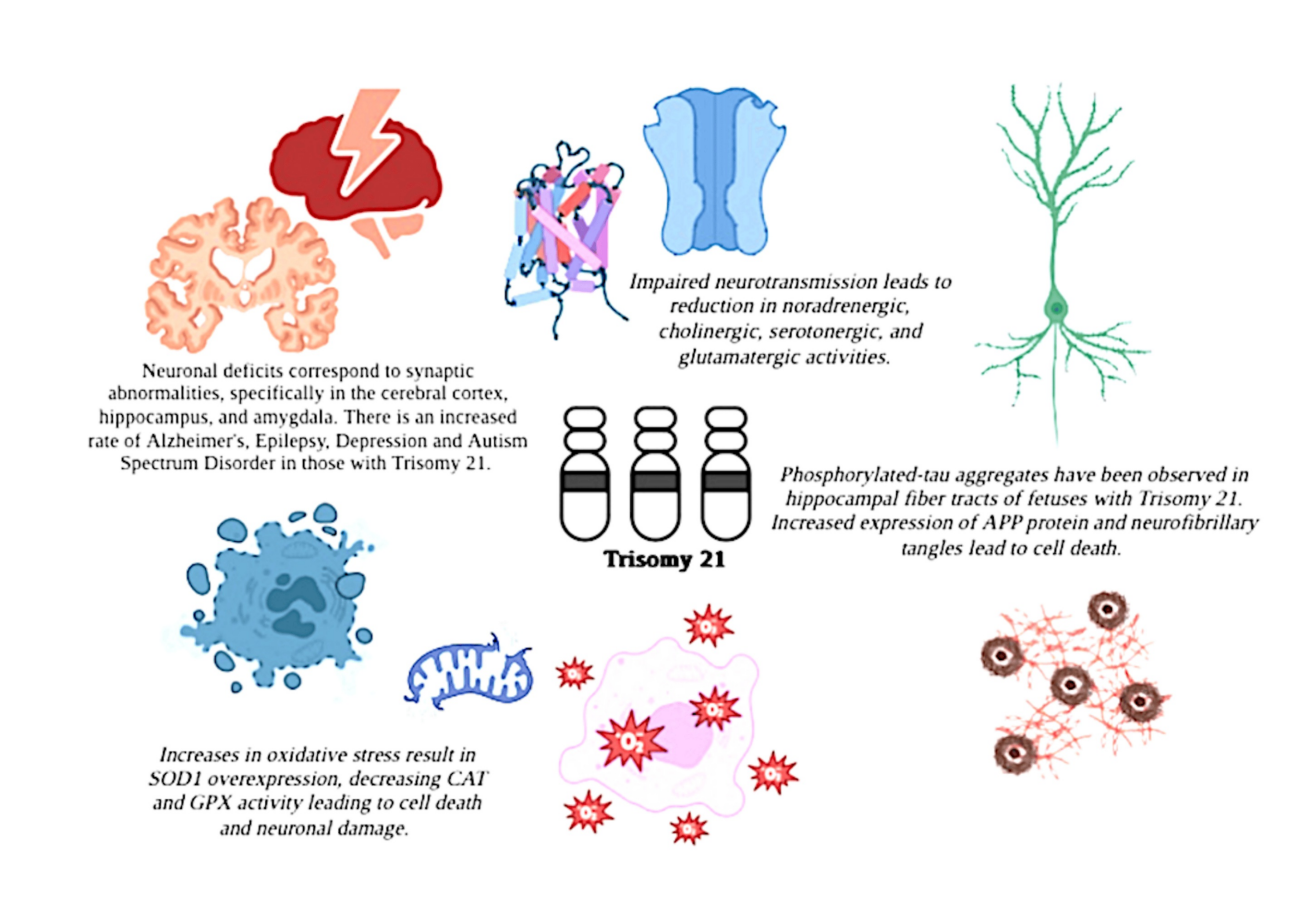
Neuronal deficits in Down syndrome Image Credits: Nidhi Chawla

Down syndrome (DS) is the most common congenital abnormality and the number one cause of genetic intellectual disability. It is well-known for its chromosomal component, the trisomy of human chromosome 21 (Hsa21) [1]. Despite the prevalence of DS, the relationship between the trisomy and the associated phenotypes is unclear. The ambiguous correlation between the genetic pathogenesis that causes DS and the resulting impairments is due in part to the broad spectrum of cognitive and intellectual deficits that can manifest with DS. The varying degrees of deficits and trisomy 21 expression make it difficult to determine the specific causes of these deficits; however, several genetic causes and environmental exposures have been linked to the establishment of trisomy 21 [2]. DS can present with cognitive and motor deficits, epilepsy, and comorbidities such as autism spectrum disorder, depression, and an elevated risk for conditions like Alzheimer's disease (AD). Trisomy 21 impairs neurodevelopment by decreasing neurotransmission, neurogenesis, and synaptic plasticity, ultimately resulting in neurodegeneration, leading to increased oxidative stress, neuronal cell death, and amyloid pathology [3]. This review aims to discuss neurodevelopmental issues that arise related to the pathophysiology found in DS. We will further discuss the correlation between morphological changes in the neurotransmitter pathways and the pathological changes that arise in sensory, cognitive, and motor development. Figure 1 describes many of the neuronal deficits seen in DS.

## Review

Neurostructural and synaptic changes in the pathophysiology of Down syndrome

Neurostructural Abnormalities

The gray and white matter ratio within the cortical tissues in those with DS is lower than that in healthy cortical tissue. This reduction in gray matter is due to the decrease in the thickness of the cortex [1,4]. Furthermore, disorganized lamination and delayed pyramidal cell growth have been seen up to 44 weeks gestational age in DS individuals but only 28 weeks in neurotypically developing fetuses [5]. Damage to the cerebral cortex results in functional deficits in coordination, reasoning, thought, and decision-making.

Similar to the progression of dementia, the progression of DS also leads to degeneration in the amygdala [1,6,7]. The amygdala is the major regulator of emotions, and it helps explain the altered dispositions often found in individuals with DS [6]. With compensation for age-related factors, research has shown that there is a distinct reduction in the volume of the amygdala in DS patients, with a larger decrease in those presenting with dementia-like symptoms [6,8]. Significant differences have been found between the brains of DS and wild-type (WT) mice in the average volume of the hippocampal commissures, including the soma, dendrites, and axon [1]. There is a greater loss of volume in the hippocampus when compared to the amygdala of those with DS [7]. When comparing the volumes of the hippocampus and amygdala in DS patients with and without dementia, the amygdala and hippocampal volumes were reduced in both regions to a greater extent in the DS patients with dementia than in the DS patients without dementia [4,6,7]. Furthermore, when the basal forebrain was examined in patients with DS, research revealed a decrease in the size, volume, and density of neuronal bodies in this area [9,10].

Synaptic Pathology

DS is associated with many neuronal deficits and corresponding synaptic abnormalities [11,12]. Through neuronal transmission, there is evidence of degeneration and disorganization, especially within the cerebral cortex, hippocampus, and amygdala [1,11,12].

Hippocampal axons show impeded growth, resulting in synaptic plasticity loss [1,11,12]. Abnormal dendrites found within the hippocampus are the proposed reason for altered synaptic plasticity in the neuronal branching patterns and network [7,12]. The neuronal densities found within the brains of those afflicted with DS not only affect morphological components but also translate to the functional changes that are observed [7,11]. Long-term potentiation (LTP) is the standard method of observing synaptic plasticity within the brain [1,7]. Alterations in LTP result in functional deficits in both learning and memory and ultimately lead to an array of neurological disorders. The brains of those with DS have shown a decrease in LTP, which correlates with the reduction in both the neuronal networks and the branching patterns of the axonal and dendritic segments [4,11,12]. However, LTP has been shown to normalize by inhibiting dual-specificity tyrosine-phosphorylation-regulated kinase 1A (*Dyrk1a*) with epigallocatechin 3-gallate, a polyphenol, in Ts65Dn mice [13].

The DS population has a distinct correlation between abnormal synaptic plasticity and learning and memory impairments [3]. Defects in the structure and function of dendritic spines can lead to both synaptic and circuit alterations, which may cause cognitive impairment, as seen in DS [14]. In a study by Benavides-Piccione et al., cognitive deficits in DS have been linked to structural changes in dendritic spines, function, and development [15]. Thrombospondins (TSP), which are regulated by astrocytes, play a crucial role in dendritic spine morphology and typical spine development. Decreased synaptic activity and morphology have been linked to abnormal astrocyte function [16,17]. Garcia et al. observed marked deficits in TSP protein expression in DS brains, but restoration of TSP1 levels prevented DS astrocyte-mediated spine and synaptic alterations [17]. This suggests that TSP1 may be a potential therapeutic target for treating spine and synaptic dysfunction in DS.

Fetuses with DS have displayed increased astrocytes in the frontal lobe at 18 to 20 gestational weeks, but the astrocyte count drops within the first year after birth [17,18]. This decrease in astroglial processes occurs alongside an increase in immature astrocytes, which are unable to provide adequate response to injury in the central nervous system (CNS) [18,19]. The decrease in mature astrocytes and increase in immature astrocytes contribute to astrogliosis, marking CNS damage and frequently associated with pathology in DS brains [19,20].

As seen in DS, alterations to such highly regulated processes can affect neuronal migration and alter cortical development [21-23]. Baburamani et al. (2020) examined fetal brains both with and without DS and found deviations in cortical growth in fetuses with DS [22]. This finding suggests that the altered radial glia impacted migratory signals, leading to cortical disturbances [22]. Some DS brains displayed various neuropathology, including white matter gliosis, hypoxic-ischemic encephalopathy, and peri-vascular/ventricular hemorrhages. Additionally, the cortical plate and ventricular zone of the fetal brains with DS had sparse cellular patterns, which may be caused by disruptions in glial development [22]. However, some DS fetal brains did not show neuropathology, and more studies are needed in this regard. The results of this study also indicated that, from 15 to 24 gestational weeks, the DS cases expressed markedly less SOX2, a progenitor cell marker, compared to age-matched controls [22]. Decreased levels of SOX2 may demonstrate a reduced neuron count, as the protein is necessary to allow for neuronal precursor self-renewal [2,21,24].

Neurotransmitter alterations in Down syndrome

Neurotransmitters are essential for neuronal communication and maintenance of a normal state. In DS, impaired gamma-aminobutyric acid (GABA), cholinergic, and glutaminergic neurons are keys to understanding the altered synaptic plasticity and eventual behavioral and cognitive deficits [25].

GABAergic

Neurotransmitter communication is altered during the progression of DS. In particular, the GABA system found in the dentate gyrus is affected [1,11]. Research has shown alterations in both synaptic and molecular properties with a concomitant increase in inhibitory postsynaptic currents (IPSC) in mouse models with DS genes [11]. The increase in IPSCs could amount to the increased presynaptic release of GABA that is found to occur in DS brains [2]. The over-inhibition present in the DS synapses results in neurochemical and structural immaturity of the brain [26]. Altered inhibition has been found specifically in cortical layers II and IV. The increases in neuronal inhibition may be the reason for the cognitive deficits that are accompanied in DS patients. A combination of increased GABA at the synapse is paralleled with the spontaneous increase in IPSCs in pyramidal neurons of the cortex [25]. One study measured decreased temporal lobe levels of GABA in children with DS, while others observed no decrease in hippocampal GABAergic markers in children [27-29]. More data are needed to further understand these results.

When observing synaptic plasticity, the prognosis of DS is more noticeable from the late infancy period onwards because both the dendritic branching and length definitively begin to shorten at six months of age [2]. The decrease in branching patterns found in the hippocampal dendrites of DS brains can be accounted for by the neurotransmitter changes [7,11]. This alteration is indicative of impairment in the neuronal network, which directly impacts an individual's ability to process information effectively [2]. Therefore, from childhood to adulthood, there is a decline in DS individuals' memory and learning abilities [2].

Cholinergic

In the basal forebrain of DS individuals, there is significant neurodegeneration of cholinergic neurons. This may be responsible for the impairments found in learning, attention, and memory. Ts65Dn mouse populations have been studied, and a distinct correlation was made between the densities of the basal forebrain and attention-based tasks [30]. Because mice demonstrating DS symptoms had lower densities of cholinergic neurons, there is research to extrapolate the connection between learning deficits and the role of cholinergic neurons in the brain of those with DS [9,10,30]. Maternal choline supplementation has been shown to be a safe and effective neuroprotective measure for those carrying fetuses with DS [9,10]. In these participants, it was found that choline supplementation improved spatial mapping and increased the overall number, density, and size of the basal forebrain cholinergic neurons [9,10].

Glutaminergic

Aside from GABAergic and cholinergic neurons, literature also addresses the role of glutaminergic neurons in DS patients. The metabotropic glutamate receptor (mGluR) is upregulated in DS subjects [9,10,31]. This observation was further supported by immunohistochemistry analysis that found both mGluR5 to be upregulated, and its role in the metabolism of amyloid precursor protein (APP) was confirmed [10,31]. AD is found to have a strong correlation with the pathology found in DS [10]. Glutaminergic projections in the cerebral cortex are found to be reduced in patients who are diagnosed with both DS and AD [31]. Glutamate deficiencies have been connected to the hippocampal N-methyl-D-aspartate receptor 1, and there is an associated downregulation of protein expression levels in the DS subjects [31,32]. These disruptions can be attributed to the deficits found in synaptic plasticity in the hippocampus, ultimately resulting in the behavioral changes found in individuals with DS [10,31,32].

In one study, no correlation was observed between hippocampal glutamate concentrations and cognitive decline [33]. In others, magnetic resonance spectra identified significantly low glutamate concentration in the hippocampus and downregulation of N-methyl-D-aspartate receptor1 in Ts2 mice [32]. The findings of glutamate are thought to be associated with impairments in synaptic plasticity; however, it is important to continue investigating glutamatergic dysfunction and its role in the hippocampus [32].

Characterizing neuronal abnormalities in Down syndrome with nuclear magnetic resonance (NMR)

NMR remains a powerful noninvasive tool for molecularly characterizing DS in live patients. Metabolites from the urine and plasma of maternal subjects who are pregnant with DS fetuses have been isolated, and NMR studies have confirmed the presence of certain types of metabolites [34]. However, although NMR has identified the presence of metabolites, the characterization of these neurotransmitters has not been well studied.

Another NMR study in a DS mouse model found smaller neurospheres in the embryonic brains of the Ts1Cje mouse model of DS compared to control mice [35]. In addition, their metabolic profile showed disruption of the utilization of glucose-6-phosphate, which suggests inefficiency regarding energy production and may account for cognitive dysfunction [35]. Glucose production and utilization through glucose-6-phosphate is an important energy source for the brain, and its disruption leads to decreased proliferation of neurospheres and a lack of adenosine triphosphate synthesis [35]. The study noted impaired glucose metabolism in the Ts1Cje mice neurosphere and in individuals with DS [35]. Impaired glucose metabolism has been correlated with less neurotransmitter synthesis, cognitive dysfunction, and DS in individuals with dementia [35].

When assessing adults with DS to their neurotypical counterparts, another study demonstrated that myo-inositol levels increased and N-acetyl-aspartate levels decreased as cognitive symptoms progressed. Recent work also revealed increased myo-inositol levels in the basal ganglia of children with DS and in the hippocampus and cerebellum of adults with DS [36]. Myo-inositol is a glial cell marker, and there have been reports of elevated levels of myo-inositol and astrocytic activation in response to inflammation [37]. Dysfunctional astrocytes and microglia cells interfere with neuronal excitability, causing learning and memory impairments, reactive states, and CNS inflammation [18]. These metabolites measure neuroinflammation and neuronal integrity, respectively. The increased myo-inositol levels are correlated with increased amyloid and tau plaques and are also seen with decreased N-acetyl aspartate levels. The progression of cortical thinning was associated with these findings, another link to the previously noted decreased gray and white matter in DS patients [38].

There has also been a recent wave of characterizing correlations between metabolites in DS patients. Some NMR metabolic profiles of DS have been characterized in serum plasma and urine [34,39]. Significant differences were found compared to controls, including disruptions in the Krebs cycle metabolites and differences in amino acids, formate, lactate, glucose, and creatine [36,39]. Protein magnetic resonance spectroscopy has also been used to analyze the metabolic phenotypes of children with DS [40]. In addition to disruptions to the tricarboxylic acid cycle, disruptions in methylation metabolism, the methionine cycle, glucose metabolism, and myo-inositol were found in people with DS. Children with DS were also found to have increased choline, N,N-dimethylglycine, and creatinine, as well as carnitine, homocysteine, creatinine, and dimethyl sulfone [41]. More research with NMR should be conducted to further examine the relationship between neurotransmitter dysfunction and DS progression.

Neurodevelopmental issues in Down syndrome and associated deficits

Cognitive and Learning Deficits in Down Syndrome

Among the range of neurological impairments in patients with DS, short-term and long-term memory deficits are two of the most likely dysfunctions associated with trisomy 21 [2]. The hippocampus, neocortex, basal ganglia, amygdala, and other areas of the brain are responsible for the production and retention of memories and are crucial for neurological development. Although cross-sectional and longitudinal studies have shown that both the onset and rate of memory decline in patients with DS are overestimated, the average age for decline can be established at 60 years [41]. However, there is significant inter-individual variability in memory performance, and such differences may become more pronounced with age. Some people may experience consistent decline as early as in their 50s. Others, however, may exhibit relatively intact memory functioning well into their 70s [41].

When comparing children with DS to the age-matched control group, the preschoolers' long-term memory performance in those with DS declined slightly [42]. However, when a similar study was conducted in the DS community across all spheres of life, there was a strong correlation between DS and memory decline. Adults and adolescents in the DS population have been found to have significant performance declines, struggling with memory tasks with longer delays [42]. Similar results were observed when the short-term memory abilities of teenagers and adults with DS and the age-matched control population were compared. DS individuals regularly failed to finish tasks such as digit and word recall [42]. Neuroimaging studies in DS participants revealed that the frontal gray matter and parietal white matter, associated with short-term and long-term memory, showed a decrease in volume when compared to those of the control population [42]. The results from these studies have allowed linkages to be drawn between the loss in frontal gray matter and parietal white matter in the DS population and its effect on memory impairment. Outcomes observed in DS patients are similar to animal experiments. Novel object recognition is a useful technique for researching mouse learning and memory. Following a single familiarization, WT mice preferred the novel object, whereas DS mice were unable to show object recognition. The results demonstrate memory impairments in trisomic mice [43].

It should also be noted that sensory impairments may also be correlated to negative learning and language outcomes, as well as seemingly maladaptive behaviors [44]. Children with DS are at higher risk of developing hearing issues, including hearing loss, ear infections, hypersensitivity to specific sounds, and cholesteatoma [44-46]. Although literature values vary from 38% to 78% of children with DS presenting with hearing loss, a cross-sectional study of 1088 children with DS found hearing loss present in approximately 85% of patients unilaterally, 91% bilaterally, and 75% presented with a heightened risk of hearing problems developing as age progressed [47,48]. One study used the short sensory profile, a report given by a caretaker of a child with DS, and found increased challenges in auditory processing to be linked to maladaptive behaviors [44]. Gregory et al. correlated a longer event-related potential wave frequency to children with DS, indicating a potential problem with sound discrimination [46]. Difficulties in discriminating sounds or being overly sensitive to specific sounds may lead to confusion, a lack of awareness within an environment, and behaviors that may appear maladaptive. This can include negative learning outcomes or decreased language skills that stem from hearing insufficiency. It is important to provide children with DS early intervention, early screening, and an environment that is not overly stimulating, as this may affect their short-term and long-term memories. A combination of both neurodevelopmental deficits and hearing loss likely equates to short-term and long-term memory deficits. This reasoning may also be applied to common features of impaired social assimilation, nonverbal communication, and sensory dysfunction often found in patients with DS.

*Motor Deficits in Down Syndrome*

DS children are 45% less likely to engage in physical activity than their neurotypical peers [49]. Further, the majority do not meet the national guidelines for physical activity. This increases their risk for comorbidities as they age [50]. Lack of activity may be correlated with motor deficits seen in people with DS. DS causes a reduction in gross and fine motor skills, including postural defects and issues with balance [51,52]. Studies have noted altered balance, gait, and lack of coordination in children with DS, in addition to impaired motor development [53-55]. Decreased neurons and neurotransmitters and impaired synapses have also been observed in children with DS, specifically within the frontal cortex, hippocampus, and cerebrum [5,52,56]. After observing decreased motor neurons in the DS mouse model, Watson-Scales et al. also reported decreased motor neurons in the spinal cord of humans [52]. However, it is not known whether DS impacts development because of the increase in the number of chromosome Hsa21 copies or if there are dosage-sensitive genes that increase in number.

Along with these findings, people with DS display atypical cerebellum size [57]. The mechanism of these deficits is still unknown, but *Dyrk1A* overexpression in Ts65Dn mice is thought to be involved in cerebellum development, loss of motor neurons, and motor impairment. When *Dyrk1A* expression is normalized or inhibited, it has been found that cognitive, motor dysfunction, and cerebellar structure improve, as well as inhibitory and excitatory balance [5,52,56,58].

Beqaj et al. revealed a positive correlation between fine motor skills, grip strength, and competency in functional domains, although physical, motor, and functional domains remained limited in children with DS [59]. For clarification, functional domains include motor skills applied in daily living, such as self-care and household maintenance, as well as mobility performance, like getting in and out of bed or on and off a bus. Alongside a study by Malak et al., which demonstrated delayed development of motor skills in DS patients compared to their neurotypical counterparts, the two studies may explain why most people with DS are less independent in their daily lives [55].

From an early age, DS children lag behind their peers in fundamental motor skills, complex skills, and functional domains [5,57]. A delay in motor skill development may also arise from a compensatory action of the brain, as it takes longer to overcome synaptic shortages and a smaller concentration of neurotransmitters [57]. However, in children with DS, fine motor skills are often completed faster but less accurately than their neurotypical controls [57]. This observation correlates with the finding that children with DS often have difficulty determining the appropriate allocation of strength in certain tasks and complete motor tasks with less precision. This may be due to the degradation of muscle tissue and hypotonia seen in DS patients [44,53].

Fine motor skills and grip strength predict functional performance in children and adolescents with DS [59]. In addition, the Bruininks-Oseretsky (BOT) test, the second and third test editions of gross motor development, and the Movement Assessment Battery for Children second edition have proven to be both reliable and reproducible metrics in assessing motor skills in children with DS [51]. It must be noted that the BOT-2 short form is more appropriate for research settings rather than clinical settings, as the intraclass correlation coefficient is lower than .9, the standard for clinical practice use [51]. However, these metrics may be used in further studies and create a more standard measurement of motor deficits in the future. Physical therapy, sensory integration therapy, and rehabilitation have helped manage symptoms of motor deficits, although more data are needed to corroborate the efficacy of these treatments [57,60]. Sensory integration training has also proved useful in improving motor skills [61].

Comorbidities stemming from altered developmental trajectories in Down syndrome

Alzheimer's Disease

AD is a neurological illness caused by amyloid-beta plaques and tau neurofibrillary triangles that most frequently manifests as dementia in the elderly. However, individuals with DS begin to exhibit signs of dementia at a significantly earlier age, often by the age of 40 [62]. Approximately 75% of DS patients have AD by age 60, and 90% are diagnosed by their 70s [63,64]. Although the likelihood of developing AD increases with age in the general population, this likelihood increases when hereditary disorders such as DS are present. Impaired gestational neurogenesis and deceased brain volumes have been linked to the overexpression of APP that results from trisomy 21 [65].

Fetal changes in patients with DS can be seen with changes in phosphorylated-tau and amyloid buildup, both indicators of AD. Phosphorylated-tau aggregates have been observed in hippocampal fiber tracts at 20 gestational weeks in DS fetuses and are increased in adults with DS as well [63,66]. Amyloid, which impairs mitochondrial function through oxidative buildup, can be detected in fetal endosomes and lysosomes as early as 28 weeks [62]. Compared to those with AD and without DS, those with DS and AD exhibit greater levels of intravascular amyloid accumulation, beginning in their teenage years [67]. The accumulation of amyloid and tau plaques is seen first in the striatum for AD and DS patients; however, DS patients typically have a higher plaque density [68]. When hyperphosphorylated, tau leads to neurofibrillary tangles, disrupts the neuronal cytoskeleton, and spreads to various cortical areas [69]. PET scans have been utilized in various studies on the DS population to link the development of AD and biomarkers such as neurofilament light, which is released after axonal damage [62].

When anticipating AD in DS individuals, a reduction of cerebral cortex and white matter volume in DS individuals between 35 and 40 years of age is suggestive of the onset of AD [70]. In addition, recent studies have shown amyloid pathology precedes AD symptom onset and neurodegeneration by an average of 15-20 years [71]. Recent work has also characterized pre-clinical changes in biomarkers of people with AD + DS. This finding could help detect early AD changes [72]. Although there is no known treatment for AD, early discovery of this condition can aid in managing AD with the use of several readily accessible tools, drugs, and support groups.

Epilepsy

Individuals with DS are also more likely to develop epilepsy (8.1-26%) in comparison to the general population (1.5-5%) [73]. Epileptics exhibit a biphasic onset of seizure, either the year after birth or within their fourth decade [2]. It is postulated that epilepsy is linked to aberrant neuronal stacking, dendritic dyskinesias, hypoplasia of the frontal and temporal lobes, and an excess of inhibitory interneurons [73]. Although people with DS have prominent GABAergic effects in their nervous systems, inhibition of GABAergic neurons creates an excitatory effect that results in seizures [73]. Extracellular potassium concentration rises due to inhibition of the GABA receptors [74]. This is thought to lower the firing threshold, facilitating action potentials and initiating epileptic effects. Another theory suggests that this may be induced by rebound stimulation in thalamic and cortical neurons [74]. Regression in the hippocampus brought on by epileptic mechanisms results in cognitive impairment.

Depression

Depression is the most common mental health diagnosis found in individuals with DS [74]. Psychiatric comorbidity affects 28.9% of children with DS [75]. One analysis of 6078 DS patients also demonstrated a higher prevalence of depression than in neurotypical patients [76].

Numerous variables, such as environment, serotonin deficiency, and neuronal malfunction, contribute to depression. One suspected potential cause of depression associated with DS is the prevalent social isolation and loneliness of people with DS [77]. Although this isolation may arise from impaired communication, such as hearing or vision loss, the environment has proven to be a factor. One study correlated impairment in social participation to the challenges of the physical environment, such as public transport, cultural and religious services, and community organizations, rather than the social environment itself [78]. Individuals with DS also perceive changes in routine or environment (missing a bus, change of plans) as negative, further contributing to the development of depression [74].

The hippocampus exhibits the most reduced volume when considering physiological and neuronal dysfunction. It has been proposed that the decrease in hippocampal volume is due to neuronal loss caused by a neurotoxic effect triggered by glucocorticoids because hippocampal volumes are significantly lower in patients when compared to those in remission from depression [79]. In depressed patients, a decrease in hippocampus size appears to reflect a decrease in neurogenesis, mitochondrial dysfunction, and an imbalance in the hypothalamic-pituitary-adrenal axis [79]. In these patients, the neurons in these regions show failed reorganization and less efficacy in neuronal plasticity. The DS population also lacks serotonin due to increased reuptake or decreased production, one of the key neurotransmitters for mood disorders [75]. Because of a series of interconnected neurodevelopmental dysfunctions and a reduction in hippocampal volume, people with DS are predicted to experience depression.

Autism Spectrum Disorder

There is a higher rate of autism spectrum disorder (ASD) diagnosis in people with DS, between 16% and 42%, compared to individuals with ASD in the general population, which is 1.9% [80]. ASD leads to further developmental and cognitive difficulties [80,81]. ASD is a complicated neurodevelopmental disease that frequently manifests as restricted and repetitive behaviors and interests, as well as impaired social communication [82].

Researchers employ various scales to assess the severity of these symptoms in people with DS and ASD. Such scales measure verbal and nonverbal communication, IQs, repetitive behaviors, and reciprocal interactions [81]. Individuals with DS + ASD demonstrate more repetitive behaviors than individuals with only DS [80]. People with DS + ASD also displayed inferior verbal and cognitive skills than participants with DS alone [82]. In comparison to people with DS-only symptoms, those with DS + ASD symptoms are more likely to experience skill regression, a delay in acquiring languages, and the employment of fewer phrases and sentences [82]. Those with DS + ASD also displayed increased autism diagnostic scores in all domains when compared to individuals with only DS [83]. Similar findings were made when measuring verbal skills, adaptability, lexical diversity, and syntactic complexity, as DS-only individuals outperformed DS + ASD individuals [80,84]. DS patients with ASD experienced problems with unusual eye contact, approaching social situations, social communication volume, and decreased relationship quality [82]. Interestingly, one study found social communication is weaker in children with ASD without DS than when compared to those with DS and ASD [85]. Early dual diagnostics may help properly plan for treatment and family support programs in DS patients with ASD.

## Conclusions

DS is a genetic disorder characterized by trisomy of human chromosome 21 and leads to significant neurodegeneration and impaired neurodevelopment. Neurotransmission pathways are often unbalanced in DS, leading to problems with synaptic communication, decreased LTP, and impaired dendritic function. Furthermore, degenerations in neuronal function and reductions in cortex thickness contribute to functional deficits in thought processes and motor skills. As well as prevalent AD at an early age, decreased hippocampus, forebrain, and temporal lobe volumes may also contribute to the increased depression and epilepsy rates seen in DS patients.

As seen in this review, the pathophysiology of neurodevelopmental deficits in DS is multifaceted. Nonetheless, DS follows a distinct pattern of developmental issue trajectories, varying from intellectual disability to motor deficits and further psychiatric and behavioral dysfunctions. Although no treatments exist to cure DS, specialized early intervention, physical therapy, and counseling may help people with DS adapt more and reach their full potential.
